# Lithium Springs

**Published:** 1917-04

**Authors:** Felix Von Oefele

**Affiliations:** New York


### Lithium Springs.

DR. FELIX VON OEFELE, New York.

Springs with over one-thousandth part of Lithium chloride to total solids are springs with a remarkable amount of lithium and springs above 1 per cent. of total solids are real lithium springs.

The name of lithia spring is much abused. Hendrixson is right in emphasizing that 300 liters of a certain claimed American Lithia water are necessary to get a single medicinal average dose of lithia. But anyhow the therapeutic effect of lithia dissolved in natural mineral waters is stronger than common lithia salts. And the amount of potassium salts present works in the same direction. Lithium urate is the most soluble urate. But the solubility of urate of potassium is not much less. Potassium salts are, as a rule, present in mineral waters in much larger quantities than lithium salts.

Lithium salts are calculated in different ways. The very low atomic weight causes very different figures. I will calculate lithium as lithium chloride where no other salt is mentioned.

Lithium chloride is present to total solids in the following European springs:

Bonifaciusbrunnen at Salzschlirf, 2.0%.
Baden-Baden, 1.9%.
Assmannshausen Thermal Water, 1.8%.
Koenigsbrunnen at Wildbad, Wuertemberg, 0.7%.
Baden, Switzerland, near 0.6%.
Kreuznach, different springs, near 0.5%.
Natalienquelle at Franzensbad, near 0.3%.
Kissingen, different springs, near 0.3%.
Kronenquelle at Salzbrunn, near 0.3%.
Wiesbaden, Kochbrunnen, near 0.3%.
Bilin, 0.15%.
Homburg, all the springs, 0.15%.
Nauheim, different springs, 0.15%.
Neuquelle at Franzensbad, about 0.1%.
Karlsbad, different springs, about 0.1%.
Kaiserquelle at Aachen, 0.07%.

The lithium present is also emphasized at Duerkheim, Assmannshausen, Tarasp, Selters, Ems, Marienbad, Wheal Clifford, Bad Orb, Sciacca and Salso Maggiore. A spring near Redruth in Cornwall, England, claims for 24 hours 400 kilogram chloride of lithium.

Some rocks contain lithium. The presence of 1 to 10% lithiumoxyd characterizes a rich lithium ore. Lithium and its salts are soluble with difficulty. Where the percentage of lithium to total solids is really high, the spring is submineralized as a rule.

The figures of chloride of lithium are about six times the figures of metallic lithium.

In the above European list, the following only, real lithium springs: Salzschlirf, Assmannshausen and Baden-Baden.

AMERICAN real LITHIA SPRINGS.

Ballardville Lithia Springs, Middlesex County, Massachusetts, with 0.0417% total solids including 10.0374% carbonate of lithium corresponding 90% of total solids. Spodumene and lepidolit, important lithium ores, are found in the neighborhood. Assmannshausen, Germany, claims to be the richest lithium spring of Europe and contains 0.0028% lithium salts calculated as bicarbonate to the water.

Nye Lithia Springs, two miles distant from Wytheville, Virginia, contains two claimed lithia springs and one claimed chalybeate spring. One of the lithia springs has 0.0315% total solids including 0.0109% carbonate of lithium corresponding to 40% lithium chloride to total solids and 1.5% iron and aluminium oxyde. The chalybeate spring has 0.0317% total solids including 0.0027% carbonate of lithium corresponding to almost 12% chloride to total solids and 7% iron and aluminum oxyde.

Geneva Lithia Springs, New York, has 0.3984% total solids including 8% lithium salts. ?? see below.

Harris Lithia Springs, Laurens County, South Carolina, has 0.1899% total solids with predominant calcium sudfate and 2% lithium bicarbonate.

Chadwick Lithia Wells at Cambridge Springs, Pennsylvania, contains 0.0028% lithium bicarbonate corresponding 4% total solids.

AMERICAN SPRINGS with remarkable lithium.

Springs containing from 1% to 0.1% down lithium chloride in total solids are springs with a remarkable amount of lithium. But together with other constituents, this amount of lithium may show some influence. There Cambridge Springs, Pa., Ballston Spa and Saratoga Springs, N. Y., Ashland, Oregon, and many others belong.

Hathorn Springs at Saratoga Springs, has 1.0% total solids.

Geyser Spring at Saratoga Springs, near 1.0%.

Ashland Pompadour Chief Spring, 0.95%.

The springs of Ballston Ppa show about the same relationship as the Koenigsbrunnen at Wildbad.

Ashland New Lithia Spring, 0.53%.

Ashland Old Lithia Spring, 0.44%.

Ashland Artesian Lithia Water, 0.10%.

For springs with less than 0.1% lithium chloride to total solids, it may be interesting to know the presence and amount of lithium for the completeness of the analysis. A further development of scientific balneology may also find these determinations useful for the relationship of different springs. But these small amounts of lithium can not have any influence on the therapeutic value of a spring.

### LOW MINERALIZED WATERS with VALID AMOUNT of EARTHS.

Where the carbonate of lime of low mineralized springs exceeds 0.015% or its bicarbonate 0.025% or carbonates of magnesia replace a corresponding part of this amount, springs with less than 0.1% total solids are excepted from listing with the low mineralized springs and are recorded here.

They may be usefully recommended for daily use for all the patients, where leakage of lime is the main symptom of the disturbed metabolism. This dilution with an adequate dissociation more easily replaces the deficit of the lime than a high concentration. The taste is good and refreshing. They are not contrarily indicated for healthy people, but they are harmful for old age and for atheromatosis.

### THERMAL CALCIUM CARBONATE WATERS.

Virginia Hot Springs:

Boiler Spring 108°F 0.0737% t.s. 83% earthy salts, 60% carbonates, calcium prevails; for bathing.

Soda Spring 74° F 0.0559% t. s. almost 90% earthy salts, 60% carbonates, calcium prevails; for drinking.

Spout Bath Spring 106°F 0.0580% t. s. over 80% earthy salts, 60% carbonates, almost 5% potassium salts, calcium prevails; for bathing.

Crook eloquently praises these waters without any definite indication and erroneously compares them with Arkansas Hot Springs and with Aix les Baines in France.

Healing Springs, near Hot Springs, Virginia, with four springs 85 to 88° F 97.25% of the spring gases is nitrogen (the springs of Tahonic Mountains are similar):

Old Spring 0.0656% t. s. 75% earthy salts; calcium and carbonic acid prevail.

New Spring 0.0646% t. s. over 75% earthy salts; calcium and carbonic acid prevail.

These springs have more sulfates than Hot Springs and a small amount of ammonium; they claim to be diuretic, somewhat laxative and tonic and are recommended for chronic congestion of the liver, irritability of the bladder from cystitis, enlarged prostata, in the early stages of Bright's disease and in debilitated states generally.

Warm Springs north of them belongs to the sulphur waters.

COLD WATERS with VALID AMOUNT OF EARTHS.

Eureka Springs, Caroll County, Arkansas, has sixty springs. The best known are: Crescent, Dairy, Basin, Magnetic, Harding, Little Eureka, Sweet, Grotto, Mystic, Oil, Arsenic, Cave and Cold.

The waters of Eureka Springs contain mainly carbonates of lime and magnesia with a small proportion of sulfates and chlorides and are recommended for rheumatism, skin, nervous, renal and bladder disorders, dyspepsia, hay fever and general debility.

Crescent Spring, Eureka Springs, 0.0091% t. s.

Dairy Spring, Eureka Springs, 0.0107%.

Basin Spring, Eureka Springs, 0.0119%.

Magnetic Spring, Eureka Springs, 0.0187%.

Senega Spring at Glen Springs, N. Y., 0.0215%.

Different Springs of Arkansas Hot Springs.

Gray Springs at Cambridge Springs, Pennsylvania, 0.0224%.

Glenn Spring, Tipton County, Tennessee, 0.0352% total solids including over 80% earth carbonates and 14.64 c inch free and half combined carbonic acid gas; claims antacid and mild diuretic property (Wildungen).

Mountain Valley Springs, Arkansas, 0.0353%.

Fauquier White Sulphur Springs, Fauquier County, Virginia, 0.0374% t. s. with predominant earths, almost 10% iron salts. 11 c inch carbonic acid gas and small quantities sulfohydrogen; claims alterative, tonic and diuretic properties; recommended in the various forms of dyspepsia, in intestinal disorders and in liver complaints, in dropsical affections due to renal and cardiac diseases as well as in the early stages of Bright's disease, in neurasthenia produced by overwork, anxiety or other causes, in certain female complaints, notably in menstrual disorders due to anaemia.

Fullerton Spring No. 1 at Cambridge Springs, Pennsylvania, 0.0376%.

Kickapoo Magnetic Spring, Warren County, Indiana, 0.0415%.

Arctic Spring, Trempelan County, Wisconsin, 0.0425%.

Magnesia Fountains at Cambridge Springs, Pennsylvania, about 0.0600%.

Alloüez Mineral Spring at Green Bay, Wisconsin, 0.0648%.

Salvator Mineral Spring, Brown County, Wisconsin, 0.0711%.

Bartlett Spring, Lake County, California, 0.0737%.

Fullerton Spring No. 2 at Cambridge Springs, Pennsylvania, 0.0785%.

### SUPERMINERALIZED EARTHY WATERS.

If the earthy waters are supermineralized, Magnesium waters, Calcium waters and Calcium-Magnesium waters are necessary subdivisions.

### MAGNESIA WATERS.

Within the earthy waters, the magnesia waters should be preferred for recommendation to old people, the lime waters for children and youth.

Allouez Mineral Springs appears there again. Evidently different analyses calculating monocarbonates and bicarbonateś are published.

---

**Concentrating Tubercle Bacilli in Sputum and Urine.** Wm. Krauss and J. S. Fleming of Memphis, Jour. of Lab. & Clin. Med. Shake 5 c.c. of sputum and 10 c.c. of 10% sodium chlorid solution thoroughly. Add ½ c.c. gasoline and shake till it is emulsified. Centrifuge at moderate speed till the gasoline forms a clear supernatant layer. Beneath this will form a thin scum containing the bacilli. Centrifuge 10 c.c. of urine at high speed, pour off supernatant urine. To the sediment add 10 c.c. of urine and 1 gram of sodium chlorid. Shake till the latter is dissolved, then add ½ c.c. gasoline and shake again. Centrifuge at moderate speed till a clear layer and scum separate as for sputum. The method is said to be more satisfactory than the antiforming-chloroform method. Instead of the ordinary centrifuge tube, a Babcock milk tube may be used to concentrate the scum. A trace of egg albumin on the slide aids the fixation of the smear.

---

**Skin Reaction for Pregnancy.** Ernst Engelhorn and Hermann Wintz, La Clinique, Montreal, July 1916, inoculating placenta extract, noted an inflammatory reaction in all of 70 pregnant women, from the second to the ninth month. A blank vaccination resulted negatively, as did vaccination with placenta extract in 53 non-pregnant women, with a single exception—a woman with a child 6 years old. They believe that with occasional exceptions, the reaction is positive from the 7th week to the 5th day after delivery, otherwise negative.

